# *Classiculasinensis*, a new species of basidiomycetous aquatic hyphomycetes from southwest China

**DOI:** 10.3897/mycokeys.40.23828

**Published:** 2018-09-18

**Authors:** Min Qiao, Wenjun Li, Ying Huang, Jianping Xu, Li Zhang, Zefen Yu

**Affiliations:** 1 Laboratory for Conservation and Utilization of Bio-resources, Key Laboratory for Microbial Resources of the Ministry of Education, Yunnan University, Kunming, Yunnan, 650091, P. R. China Yunnan University Kunming China; 2 School of Life Sciences, Yunnan University, No. 2 North, Kunming, Yunnan, 650091, P. R. China Yunnan University Kunming China

**Keywords:** fresh water fungi, mycoparasites, Pucciniomycotina, taxonomy

## Abstract

*Classiculasinensis*, isolated from decaying leaves from Mozigou, Chongqing Municipality, China, is described as a new species. The new species is a member of basidiomycetous aquatic hyphomycetes which represent a small proportion of all aquatic hyphomycetes. This species falls within the genus *Classicula* (Classiculaceae, Pucciniomycotina) and is closely related to *C.fluitans*, based on multiple gene sequence analyses. Morphologically, it is characterised by the apical, hyaline, obclavate or navicular conidia with several hair-like lateral appendages and by its holoblastic and monoblastic conidiogenesis, with a flat un-thickened conidiogenous locus. Clamp connections and haustorial branches were often observed in culture.

## Introduction

Aquatic hyphomycetes constitute a dominant mycoflora on submerged decaying plant debris, both in lotic and lentic systems (Khan 1987). Phylogenetically, most aquatic hyphomycetes belong to Ascomycota, with only a small percentage belonging to Basidiomycota (Shearer 2007). Most known basidiomycetous aquatic hyphomycetes have been reported from North America (Marvanová 1977, Marvanová and Barlocher 1988, 1998, 2000, Marvanová and Suberkropp 1990, Raj 1981), Australia (Shaw 1972), Asia (Hudson and Ingold 1960, Nawawi 1985, Marvanová and Bandoni 1987, Kirschner 2013) and Europe (Scheuer 2008).

There are more than 8000 known species in the Pucciniomycotina (previously Urediniomycetes) and these comprise about one-third of all described basidiomycetes (Aime et al. 2006). Classification of the Pucciniomycotina has been reviewed and revised multiple times. Based on sequences at large and small subunits of the nuclear rDNA, Aime et al. (2006) grouped them into 8 classes. More recently, two new classes, Tritirachiomycetes (Schell et al. 2011) and Spiculogloeomycetes (Wang et al. 2015), were added to Pucciniomycotina. Amongst these 10 classes in Pucciniomycotina, one class, the Classiculomycetes, contains a single order, the Classiculales, with only two monotypic fungi, *Jaculispora* H. J. Huds. & Ingold and *Classicula* R. Bauer, Begerow, Oberw. & Marvanová. As early as 1960, *Jaculispora* was erected to accommodate a single anamorphic fungal species, *J.submerse* H. J. Huds. & Ingold. The genus is characterised by having narrow and delicate conidiophores and obclavate conidia with 0–3 hair-like lateral appendages (Hudson and Ingold 1960). Later, Naiadella Marvanova & Bandoni was established with *N.fluitans* Marvanova & Bandoni (synomy of *C.fluitans*) as the type species (Marvanová and Bandoni 1987). Bauer et al. (2003) observed the basidial stage of *N.fluitans* and connected it to the teleomorphic state *Classicula*. *Classicula* was recommended over *Naiadella* because *Classicula* is the base of the higher level taxonomy (Aime et al. 2018). The conidia of *Classicula* are similar to those of *Jaculispora* in shape. *Classicula* is characterised by the production of clamped hyphae with tremelloid haustorial cells and binucleate fusoid conidia with 3–4 bristle-like lateral branches (Marvanová and Bandoni 1987). Bauer et al. (2003) defined the phylogenetic positions of genera *Jaculispora* and *Classicula* based on the small subunit of ribosomal DNA (18S rDNA). Subsequent analyses of both the 18S and the large subunit ribosomal DNA (28S rDNA) data also supported the conclusion that the two genera are closely related and both belong to class of Classiculomycetes (Schell et al. 2011).

During a study of aquatic hyphomycetes on submerged decaying leaves collected from a stream in south-western China, we encountered two fungi which resembled species in the genus *Classicula*. Combining the morphological and phylogenetic analyses, we identified that the fungi belonged to *Classicula*. In this paper, we describe these specimens as a new species and discuss its phylogenetic placement based on the combined sequences of the 18S and 28S rDNA, the internal transcribed spacer regions of rDNA (ITS 1 and 2, including the 5.8S rDNA gene) and the translation elongation factor 1-a (TEF1).

## Materials and methods

### Collection of samples, isolation and characterisation

Samples of submerged dicotyledonous plant leaves collected from a stream in Chongqing Municipality were transported to the laboratory in zip-locked plastic bags. The rotten leaves were cut to several 0.5–1.5 × 1–1.5 cm sized fragments in the laboratory and spread on to the CMA medium (20 g cornmeal, 18 g agar, 40 mg streptomycin, 30 mg ampicillin, 1000 ml distilled water). After incubation at 27 °C for about 10 days, a single conidium was isolated and cultivated on CMA in Petri plates using a sterilised toothpick under a BX51 microscope. Morphological observations were made from cultures on CMA after incubation at 27 °C for one week. Pure cultures and a permanent slide were deposited in the Herbarium of the Laboratory for Conservation and Utilization of Bio-resources, Yunnan University, Kunming, Yunnan, P.R. China (YMF; formerly Key Laboratory of Industrial Microbiology and Fermentation Technology of Yunnan). Ex-holotype living cultures were deposited in the China General Microbiological Culture Collection Center (CGMCC).

### DNA extraction, PCR and sequencing

The cultures were grown on potato dextrose agar (PDA) and incubated at 27 °C for about 10 days. Fungal mycelia were harvested and transferred to a 2.0 ml Eppendorf tube. Total DNA was extracted using a CTAB method as described by Pratibha et al. (2014).

Three regions of the nuclear ribosomal DNA gene cluster and one nuclear protein-coding genes, translation elongation factor 1a (TEF1) were amplified: Primer pairs ITS4 and ITS5 (White et al. 1990) were used to amplify the complete ITS regions (including 5.8 S); NS1 and NS8 for the 18S rDNA; and LR5 and LROR for the 28S rDNA (Vilgalys and Hester 1990). Primer pairs EF1-983F and EF1-2218R were used for amplifying the TEF1 gene (Rehner and Buckley 2005). PCR amplifications were performed using the methods described previously (Wang et al. 2014). The PCR products were then sent to the Beijing Tsingke Biotechnology Co. of China, Ltd and sequenced on both strands with the same primers that were used for amplification.

### Sequence alignment and phylogenetic analysis

Preliminary BLAST searches with 18S and 28S rDNA gene sequences of the new isolates indicated that they had a close phylogenetic relationship with sequences from the genera *Jaculiapora* and Naiadella (Classicula). Based on the phylogenetic positions of the two genera, we downloaded 18S, 28S, ITS and TEF1 sequences of representative species of 8 class within Pucciniomycotina, but Cryptomycocolacomycetes and Spiculogloeomycetes were not included as Cryptomycocolacomycetes only includes two known species, *Cryptomycocolaxabnormis* and *Colacosiphonfiliformis* and only 18S rDNA of two species are available. Spiculogloeomycetes only comprises yeast and yeast-like species, which has an affinity to Mixiomycetes within Pucciniomycotina. Based on our main aim of identifying new hyphomycetes species within Classiculomycetes, another 8 classes were chosen to carry out phylogenetic analysis. Four sequences of each representative strain of 8 classes were combined with those from our own cultures. (see Table 1 for all GenBank accession numbers).

Raw sequences were aligned using CLUSTAL W 1.6 (Thompson et al. 1994); then manually adjusted to minimise the number of uninformative gaps and to improve alignments using MEGA 6.06 (Kumar et al. 2012). Ambiguously aligned regions were excluded from downstream analyses. Missing data at the 5'- and 3'-end of partial sequences were coded by a ‘?’. To select the most appropriate model of sequence evolution, JMODEL TEST 2.1.1 was run for each gene (ITS, TEF1, 18S, 28S) and the GTR þ I þ G model was chosen according to the Akaike information criterion (AIC). Before phylogenetic analysis, the ITS, TEF1, 18S and 28S matrices were concatenated with BIOEDIT 7.5.0.3. The tree construction procedure was performed in MrBAYES 3.2 (Ronquist et al. 2012). Maximum likelihood was performed with MEGA 6.06. *Auricularia* sp. and *Coprinuscomatus* of Agaricomycotina were used as outgroups. Phylogenetic trees were imported into FIGURETREE 1.4.2 and exported as SVG vector graphics for Figure assembly in ADOBE ILLUSTRATOR CS6. The phylogenetic analyses of different datasets were performed using Bayesian and maximum likelihood algorithms.

**Table 1.** The species used in the phylogenetic analyses. Also included in the Table are the representative isolate name of each species and the GenBank accession numbers for each of the four analysed gene fragments of each isolate.

Class	Species	Isolate No.	GenBank accession No.				Reference
			ITS	28S	18S	TEF	
Agaricostilbomycetes	* Bensingtonia changbaiensis *	AS 2.2310	AY233339	AY233339	AY233339	KJ707751	Wang et al. 2003; 2015
	* Agaricostilbum hyphaenes *	CBS 7811	AF444553	AF177406	AY665775	KJ707749	Scorzetti et al. 2002; Wang et al. 2015
	* Chionosphaera apobasidialis *	CBS 7430	AF444599	AF177407	U77662	KJ707883	Scorzetti et al. 2002; Wang et al. 2015
	* Bensingtonia ciliata *	AS 2.1945	AF444563	AF189887	D38234	KF706486	Scorzetti et al. 2002; Wang et al. 2015
	* Kurtzmanomyces insolitus *	JCM 10409	AF444594	AF177408	KJ708424	KJ707893	Scorzetti et al. 2002; Wang et al. 2015
	* Sporobolomyces sasicola *	AS 2.1933	AF444548	AF177412	AB021688	KJ707900	Scorzetti et al. 2002; Wang et al. 2015
	* Mycogloea nipponica *	CBS 11308	KJ778629	KJ708456	KJ708370	KJ707882	Wang et al. 2015
	* Sterigmatomyces elviae *	JCM 1602	AB038053	KP216512	KP216516	KJ707852	Wang et al. 2015
	* Kondoa aeria *	CBS 8352	AF444562	AF189901	KJ708417	KJ707905	Scorzetti et al. 2002
Cystobasidiomycetes	*Bannoa* sp.	MP 3490	DQ631900	DQ631898	DQ631899	DQ631902	Matheny et al. 2006
	* Naohidea sebacea *	CBS 8477	DQ911616	DQ831020	KP216515	KF706487	Wang et al. 2015
	* Sporobolomyces coprosmae *	JCM 8772	AF444578	AF189980	D66880	KJ707798	Scorzetti et al. 2002
	* Sakaguchia dacryoidea *	JCM 3795	AF444597	AF189972	D13459	KP216514	Scorzetti et al. 2002
	* Sporobolomyces bischofiae *	JCM 10338	AB035721	AB082572	AB035721	KJ707777	Hamamoto et al. 2002
	* Rhodotorula armeniaca *	JCM 8977	AF444523	AF189920	AB126644	KJ707762	Scorzetti et al. 2002; Wang et al. 2015
	* Occultifur externus *	JCM 10725	AF444567	AF189910	AB055193	KJ707829	Scorzetti et al. 2002; Wang et al. 2015
	* Cyrenella elegans *	CBS 274.82	KJ778626	KJ708454	KJ708360	KJ707830	Wang et al. 2015
	* Erythrobasidium hasegawianum *	AS 2.1923	AF444522	AF189899	D12803	KJ707776	Scorzetti et al. 2002; Wang et al. 2015
Pucciniomycetes	* Chrysomyxa arctostaphyli *	CFB22246	DQ200930	AY700192	AY657009	DQ435789	Matheny et al. 2007
	* Endocronartium harknessii *	CFB22250	DQ206982	AY700193	AY665785	DQ234567	Matthias et al. 2004
	* Helicobasidium mompa *	CBS 278.51	AY292429	AY254179	U77064	EF100614	Matthias et al. 2004
	* Platygloea disciformis *	IFO32431	DQ234556	AY629314	DQ234563	DQ056288	Matheny et al. 2007
	* Puccinia graminis tritici *	CRL75-36-700-3/ECS	AF468044	AF522177	AY125409	XM_003333024	Weber et al. 2003
	* Insolibasidium deformans *	TDB183-1	–	AF522169	AY123292	–	Wang et al. 2015
	* Septobasidium canescens *	DUKE:DAH(323)	DQ241446	DQ241479	DQ241410	–	Henket al. 2007
Tritirachiomycetes	* Tritirachium oryzae *	CBS 164.67	GQ329853	KF258732	JF779647	JF779645	Schell et al. 2011
	*Tritirachium* sp.	CBS 473.93	JF779664	JF779649	JF779650	JF779651	Schell et al. 2011
	*Tritirachium* sp.	CBS 265.96	JF779668	JF779652	JF779653	-	Schell et al. 2011
Mixiomycetes	* Mixia osmundae *	CBS 9802	DQ831010	DQ831009	D14163	KJ707837	Matheny et al. 2006
Microbotryomycetes	* Leucosporidium scottii *	JCM 9052	AF444495	AF070419	X53499	KJ707788	Scorzetti et al. 2002; Wang et al. 2015
	* Sphacelotheca hydropiperis *	CBS 179.24	KJ708463	KJ708463	KJ708394	KJ707807	Wang et al. 2015
	* Microbotryum violaceum *	CBS 143.21	KJ708462	KJ708462	KJ708388	KJ707811	Wang et al. 2015
	* Sporobolomyces bannaensis *	AS 2.2285	AY274824	AY274823	KJ708405	KJ707934	Zhao et al. 2003
	* Rhodosporidium babjevae *	JCM 9279	AF444542	AF070420	AB073270	KJ707874	Scorzetti et al. 2002; Wang et al. 2015
	* Rhodotorula rosulata *	CBS 10977	EU872492	EU872490	KJ708384	KJ707854	Wang et al. 2015
Atractiellomycetes	* Helicogloea lagerheimii *	FO 36341	–	AY512849	AY124476	–	Bauer et al. 2003
	* Helicogloea variabilis *	KW 1540	–	L20282	U78043	–	Berres et al. 1995
	* Platygloea vestita *	DB 1280	–	AY512872	AY124480	–	Bauer et al. 2003
Classiculomycetes	* Classicula fluitans *	ATCC 64713	–	AY512838	AY124478	–	Schell et al. 2011
	*** Classicula sinense ***	**YMF 1.04613**	**KY548838**	**KY548836**	**KY468515**	**MG787169**	This study
	*** Classicula sinense ***	**YMF 1.04389**	**KY548837**	**KY548835**	**KY468514**	**MG787170**	This study
	* Jaculispora submersa *	CCM 8127	–	AY512853	AY124477	–	Schell et al. 2014
Agaricomycotina	*Auricularia* sp.	AFTOL-ID 676	DQ200918	AY634277	DQ234542	DQ408144	Schell et al. 2014
	* Coprinus comatus *	AFTOL-ID 626	AY854066	AY635772	AY665772	AY881026	Schell et al. 2014

## Results

### Phylogenetic analysis

**Figure 1.**
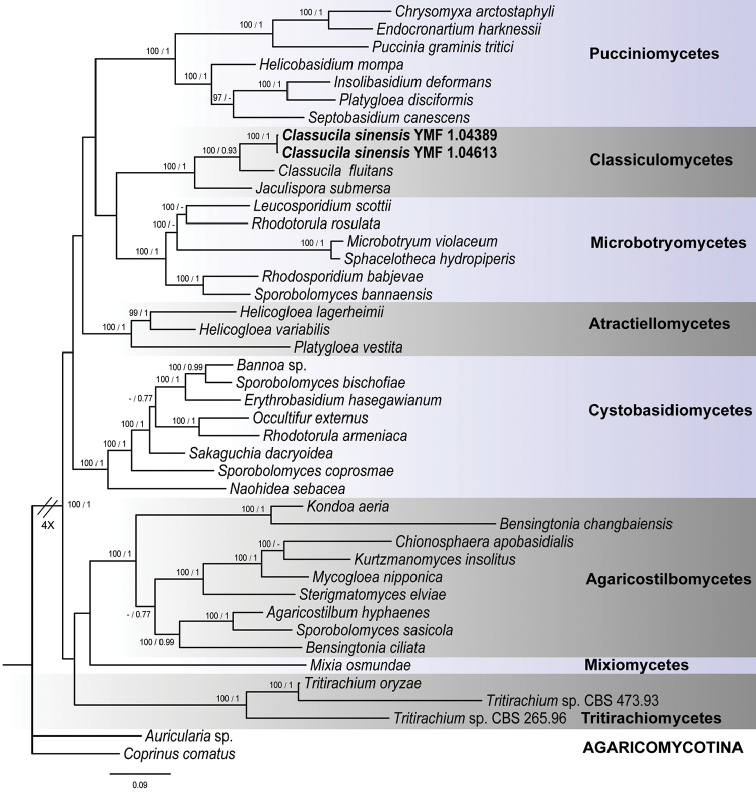
Phylogenetic tree based on Bayesian analysis of the combined ITS, TEF1, 18S and 28S rDNA sequences. *Auricularia* sp. and *Coprinuscomatus* of Agaricomycotina are used as outgroups. Clades and taxa are labelled according to Schell et al. (2011). Bayesian posterior probabilities, greater than 0.95, are given above the nodes (out of 100). Maximum likelihood bootstrap values, greater than 75%, are given below the nodes (out of 100). The scale bar shows the expected changes per site.

In our Bayesian and maximum likelihood analyses (Figure 1), our isolates representing the new species named *C.sinensis* was a sister group to *C.fluitans* and consistently had *J.submerse* as the next closest relative with a strong statistical support. The close relationship between *C.sinensis* and *C.fluitans* was supported with a posterior probability of 1.00 in the Bayesian analysis and with a bootstrap value of 0.93 in the maximum likelihood analysis. Phylogenetic relationships amongst the taxa inferred from the combined four gene sequences are in general agreement with those based on SSU rDNA and LSU rDNA D1/D2 domains by Wang et al. (2015). Although there are some minor variations in the relationships amongst the classes between the two studies, taxa within each class still formed a single clade.

BLAST searches using the complete ITS regions of our *C.sinensis* strains (YMF 1.04613 and YMF 1.04389) aligned them only to the 5.8S rDNA of a variety of uncultured fungus. There are a few ITS1 matches at about 87% sequence identity to specimens in Pucciniomycotina. Since the study of Classiculomycetes by Bauer et al. (2003) did not employ ITS sequences, we were unable to use ITS sequences for species confirmation with those in *Classicula*. Sequences of accession numbers AY512838 and AY512853 were those of 18S rDNA of *C.fluitans* R. Bauer, Begerow, Oberw. & Marvanová and *J.submerse*, respectively, but were mistaken for ITS by Wang et al. (2015).

### Taxonomy

#### Classicula sinensis

Taxon classificationFungiClassiculalesClassiculaceae

Y. Huang & Z.F. Yu sp. nov.

819813

Figure 2

##### Etymology.

Sinensis refers to the country in which this species was found.

##### Diagnosis.

***Classiculasinensis*** differs from *C.fluitans* by having fusiform conidiogenous cells growing from the hyphae directly.

##### Type.

**CHINA**. From leaves of an unidentified dicotyledonous plant submerged in a stream, Chongqing Municipality, Mozigou, 29°25'38"N, 107°24'19"E, ca. 750 m elev. Oct 2014, ZeFen Yu, YMF 1.04613–holotype[live culture], YMFT1.04613 [dried specimen], CGMCC–3.18938–ex-type culture. Other strain: YMF 1.04389, CGMCC–3.18937, Chongqing Municipality, Mozigou, 29°28'N, 107°25'E, ca. 750 m elev.

##### Description.

Colonies on CMA reach about 10 mm diameter after incubating for 7 days at 27 °C. Colony effuse, mycelium partly superficial, partly immersed in substratum, composed of hyaline, branched, thin-walled, septate, smooth, binucleated hyphae, 1.5–4.8 µm wide, often 1.8–2.7 µm wide. Clamp connection and haustorial branches on hyphae present. Haustorial branches with basal clamps, tapering distally or obclavate, 9–14.2 (–16.5) µm long, 1.2–2.6 µm wide, one or two terminal filaments of 3–8.5 × 1.3 µm located on the top of it. Conidiophores absent. Conidiogenous cells fusiform, monoblastic, 7.5–11×2–2.8 µm, attaching directly on the hyphae, solitary or in aggregates of two. Conidia solitary, acrogenous, navicular or obclavate, attenuating upwards, 25–38 (–42) µm long, 3.8–6.2 µm wide, 1.3–3.4 µm wide at the truncate base, (0–) 2–5 (–7) septa appear in those conidia without cytoplasm, with 1–5 (mainly 3–4) lateral appendages, attaching to the upper part of conidia, opposite or verticillate, filiform, smooth, divergent, pendulous or straight (–7) 13–21 (–25) µm long, 0.8–1.2 µm wide, 0–2(–3) septate. Occasionally, 1(–2) appendages also arise from apex of the main axis. Sometimes clamp connections appear at the top of conidia.

**Figure 2.**
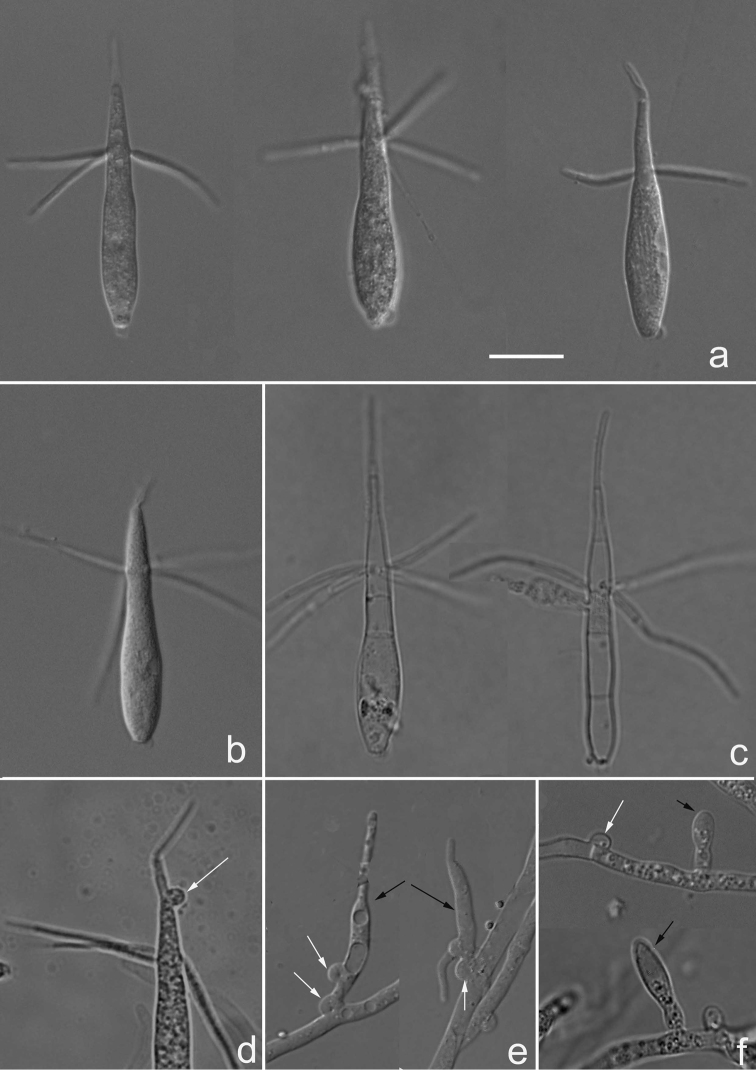
Microscopic features of *Classiculasinensis* (holotype YMF 1.04613). **a, b** Conidia **c** empty conidia **d** clamp connection on conidia **e** Haustorial branches with basal clamps on hyphae **f** Conidiogenous cells (black arrow) and clamp connection on hyphae (white arrow). Scale bar: 10 µm **(a–f).**

## Discussion

*Classicula* is phylogenetically related to *Jaculispora* and morphologically similar to the latter. When *Jaculispora* was established, Hudson and Ingold (1960) did not mention clamp connections or haustorial cells. Later, Matsushima (1987) observed clamps on hyphae from *J.submersa* (isolate MFC 12864), but did not see haustorial cells. Bauer et al. (2003) reported that *J.submersa* also presented tremelloid haustorial cells similar to *C.flutitans* and both species have septal-spore architecture surrounded by microbodies. Further phylogenetic analysis inferred from the 18S rDNA gene revealed that the two species belonged to the family Classiculaceae of Urediniomycetes. In fact, both *Classicula* and *Jaculispora* are very similar in having navicular conidia with 3–4 distal setose branches. However, conidiogenous cells of *Classicula* are discrete fusiform, differentiated obviously and those of *Jaculispora* are integrated. Conidiogenous cells of *C.sinensis* are also integrated, but in the analysis of concatenated dataset of four sequences, *C.sinensis* and *C.flutitans* formed a well-supported clade separated from *Jaculispora*, so we treated our strains as a member of the genus *Classicula*.

*C.fluitans* is similar to *C.sinensis* in having haustorial branches and obclavate or navicular conidia with hair-like lateral and apex appendages. However, their conidiophores and conidiogenous cells were totally different. First, *C.sinensis* has no conidiophore and its conidiogenous cells grow from hyphae directly, while conidiophores of *C.fluitans* are determinate, micronematous to semi-macronematous. Second, typical conidiogenous cells of *C.fluitans* are discrete fusiform formed successively, clamped basally, but *C.sinensis* has no clamps at the base of conidiogenous cells and conidiogenous cells of *C.sinensis* are integrated, which resemble that of *Jaculispora*. Besides the main differences described above, conidia of *C.fluitans* are shorter and wider [(18–)25– 32(–45) × (4–)5–6.5(–9)] than those of *C.sinensis*, lateral branches of *C.fluitans* are 2–3, while 4 lateral branches often appear in *C.sinensis*. Furthermore, coralloid structures were interpreted as appressoria in *C.fluitans* but were not observed in *C.sinensis* (Bauer et al. 2003).

*C.sinensis* is similar to *J.submersa* in conidia form, but conidia of the latter grow on the tip of long micronematous conidiophores, while that of *C.sinensis* grow from conidiogenous cells directly produced on hyphae. Besides, conidia of *J.submersa* are longer than those of *C.sinensis* (type strain: 35–55 × 5–7, MFC-12864:35–56 × 4–6 µm). Septa of conidia without cytoplasm were not mentioned in the type strain of *J.submerse*. In strain MFC-12864, there is a septum obscurely presented at the attenuated part, while conidia of *C.sinensis* have 3–4 septa after cytoplasm drained out of the conidia.

A combination of morphological and molecular characters was used to establish *C.sinensis*. Conidiogenous cells of *C.sinensis* and *C.flutitans* were sufficiently different to support the molecular data and to suggest the new species. This situation has not been observed often in other fungi of the same genus, thus more isolates belonging to Classiculomycetes are needed to circumscribe genus characteristics of *Classicula* better and in more detail.

## Supplementary Material

XML Treatment for Classicula sinensis
